# Supplementary material for: KCC2 overexpression prevents the paradoxical seizure-promoting action of somatic inhibition
Source: Nat Commun. 2019 Mar 15;10:1225. doi: 10.1038/s41467-019-08933-4 (PMC6420604; doi:10.1038/s41467-019-08933-4)
Supplement: Supplementary file 1 — Supplementary Information [file 41467_2019_8933_MOESM1_ESM.pdf]

Supplementary information

KCC2 overexpression prevents the paradoxical seizure-promoting action of somatic inhibition

Magloire et al.

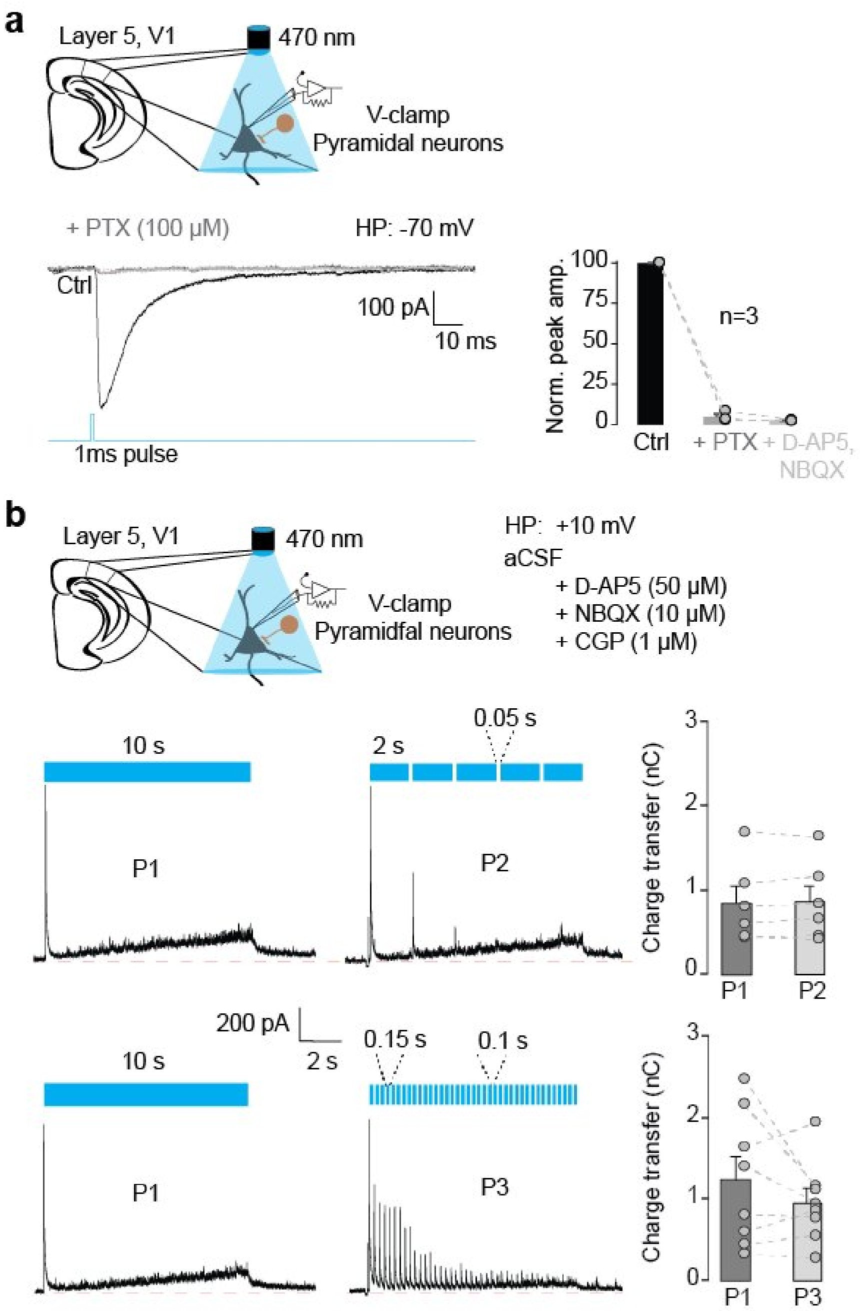

Supplementary Figure 1. Optogenetic activation of ChR2-expressing PV+ interneurons in V1.

(a) Currents evoked by blue light pulses in V1 layer 5 pyramidal neurons are abolished by the $GABA_A$ receptor antagonist picrotoxin (PTX; n = 3 cells). HP: holding potential, V-Clamp: voltage clamp

(b) $GABA_A$ receptor-mediated currents in pyramidal neurons induced by 3 different optogenetic stimulation protocols (P1: continuous 10 s stimulation; P2: five 2-s-long pulses delivered with 50 ms intervals; P3: 10 s stimulation with 150 ms-long pulses separated by 100 ms intervals). Continuous and intermittent photostimulation produces similar $GABA_A$ receptor-mediated charge transfer (P1 vs. P2: n = 6 cells; P1 vs P3: 8 cells; $p > 0.05$, paired t-test).

Error bars represent s.e.m. Source data are provided as a Source Data file.

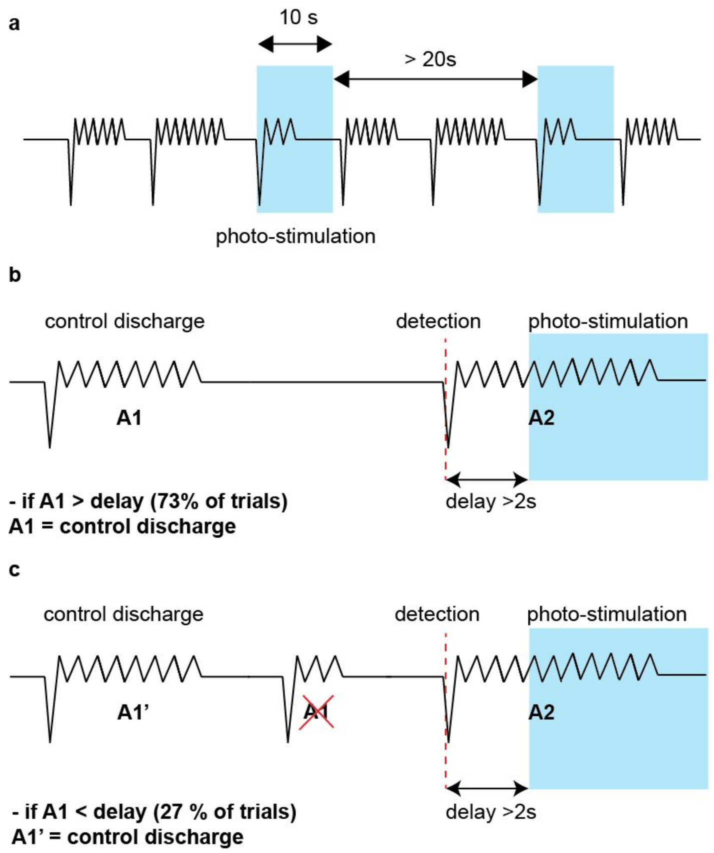

Fig. 2. Experimental protocol and analysis of delayed laser activation.

(a) Experimental protocol. Laser activation periods were separated by at least 20 s to allow data acquisition for 10 s before (baseline) and after the light pulse. The last ictal discharge during the baseline period preceding the one which was used to trigger laser activation was used as a control. On some occasions the control ictal discharges and discharges that triggered laser activation were separated by more than 10 s.

(b) In the majority of trials (73%), the duration of the discharge that was used to trigger laser activation was compared to that of the immediately preceding control discharge, if it was longer than the photostimulation delay.

(c) In the other 27 % of trials, the immediately preceding discharge was shorter than the photostimulation delay. In these cases, the nearest (within 30 s) preceding discharge of sufficient length was used as the control.

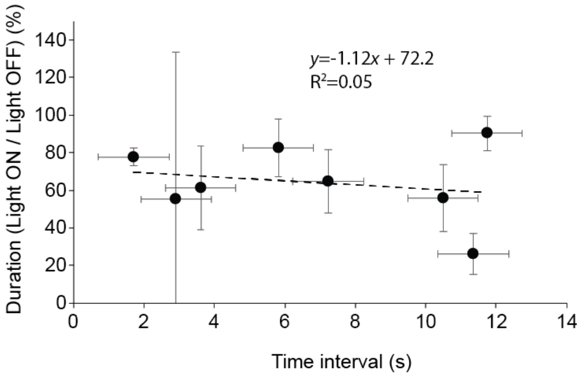

Supplementary Figure 3. Seizure suppression by photo-depolarization of ChR2-expressing PV+ interneurons is independent of the time interval between seizures.

Data points represent individual animals; error bars represent s.e.m.

Source data are provided as a Source Data file.

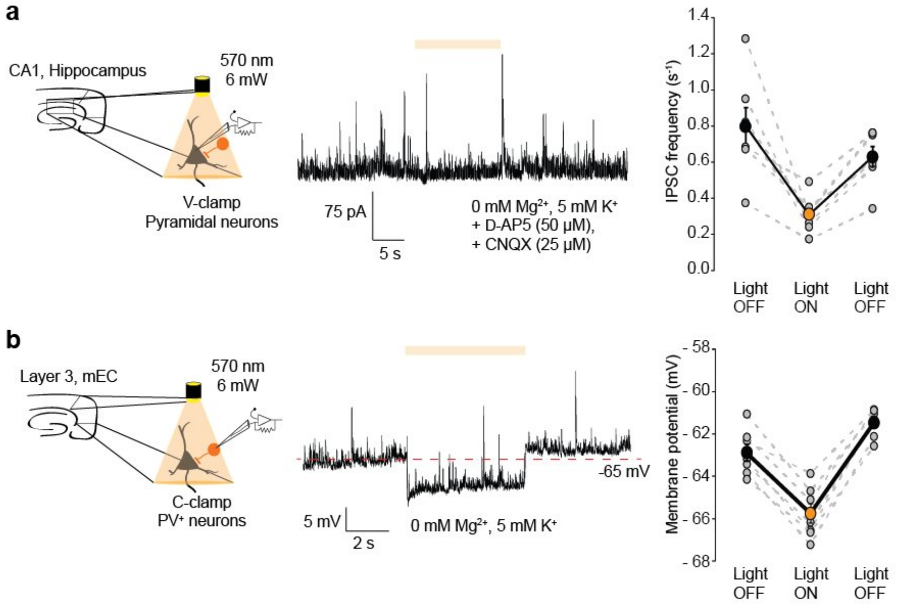

Supplementary Figure 4. Optogenetic hyperpolarization of PV+ interneurons expressing Arch3.0.

(a) Continuous yellow illumination of a slice with Arch3.0-expressing PV+ cells reduces the frequency of spontaneous $GABA_A$ receptor-mediated postsynaptic currents recorded in a pyramidal neuron.

(b) Whole-cell current-clamp recording demonstrating photostimulation-induced hyperpolarization of a PV+ interneuron expressing Arch3.0. C-Clamp: current clamp. mEC: medial Entorhinal Cortex.

Error bars represent s.e.m. Source data are provided as a Source Data file.

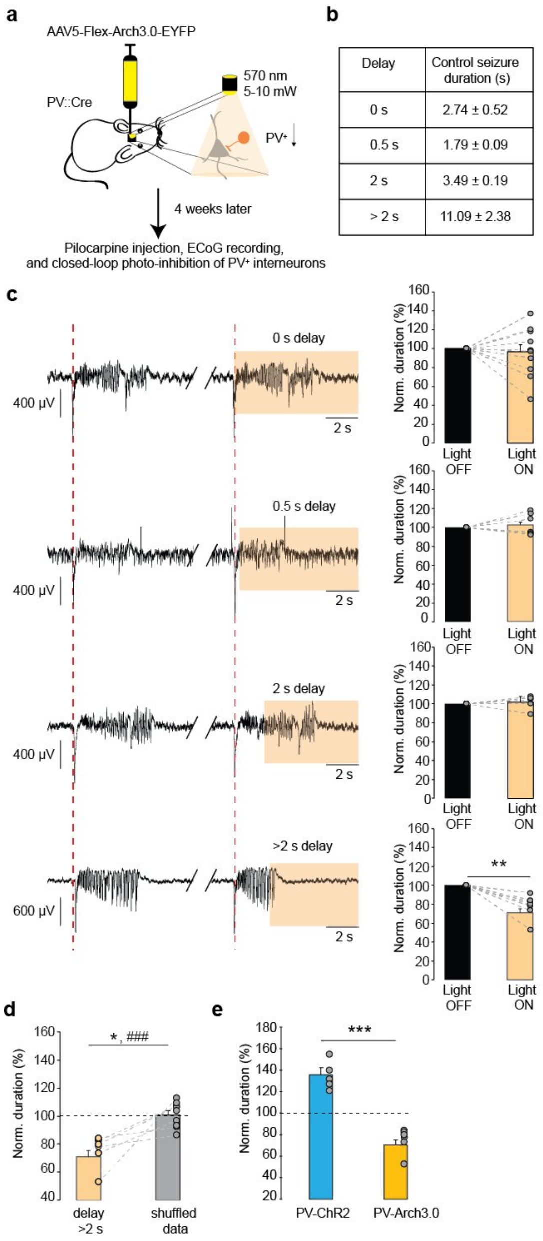

Delay	Control seizure duration (s)
0 s	2.74 ± 0.52
0.5 s	1.79 ± 0.09
2 s	3.49 ± 0.19
> 2 s	11.09 ± 2.38

Supplementary Figure 5. Photo-hyperpolarization of Arch3.0-expressing PV+ interneurons reduces the duration of electrographic seizures

(a) *In vivo* experimental schematic.

(b) Mean durations of control ictal discharges in experiments with different delays.

(c) Closed-loop photo-hyperpolarization of PV+ neurons reduces the duration of ictal discharges when delayed by more than 2 s (n = 11, 7, 5 and 6 mice for 0, 0.5, 2 and >2 s delays respectively, paired t-test). Sample traces illustrate pairs of consecutive seizures without and with laser activation (intervening periods between seizures are omitted; yellow rectangles indicate laser on).

(d) The effect of delayed photo-hyperpolarization of PV+ interneurons compared to reshuffled data (* $p < 0.05$ paired t-test, n = 6; ### $p < 0.001$, unpaired t-test, n = 9, includes data from additional mice injected with the Arch3.0 construct).

(e) Summary graph comparing the effects of delayed photo-depolarization (PV-ChR2, *blue*, n = 5) and photo-hyperpolarization (PV-Arch3.0, *yellow*, n = 7) of PV+ interneurons, unpaired t-test.

* $p < 0.05$, ** $p < 0.01$, *** $p < 0.001$; error bars represent s.e.m. Source data are provided as a Source Data file.

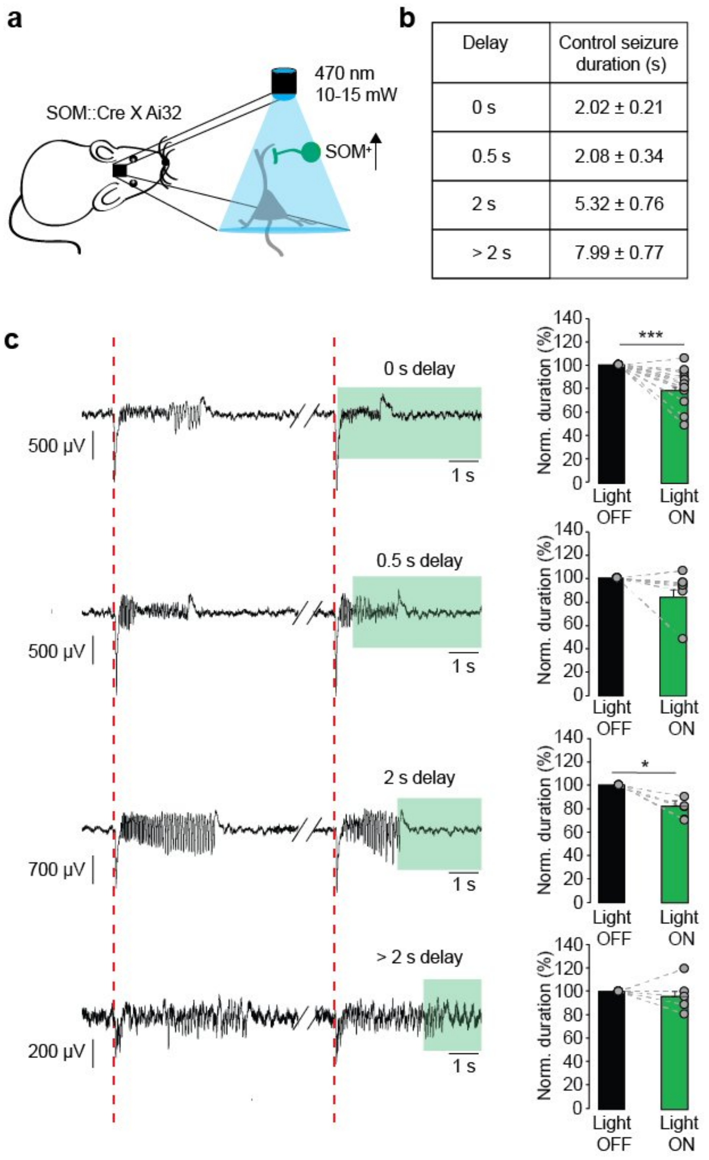

Delay	Control seizure duration (s)
0 s	2.02 ± 0.21
0.5 s	2.08 ± 0.34
2 s	5.32 ± 0.76
> 2 s	7.99 ± 0.77

Supplementary Figure 6. Closed-loop photostimulation of ChR2-expressing SOM+ interneurons has no seizure-promoting action.

(a) *In vivo* experimental schematic.

(b) Mean durations of control ictal discharges in experiments with different delays.

(c) Closed-loop photo-depolarization of SOM+ interneurons with 0 s (n = 13), 0.5 s (n = 8), 2 s (n = 5), >2 s (n = 6) delays. Sample traces illustrate pairs of consecutive seizures without and with laser activation (intervening periods between seizures are omitted; green rectangles indicate laser on).

Paired t-test, * $p < 0.05$, ** $p < 0.01$, *** $p < 0.001$; error bars represent s.e.m. Source data are provided as a Source Data file.

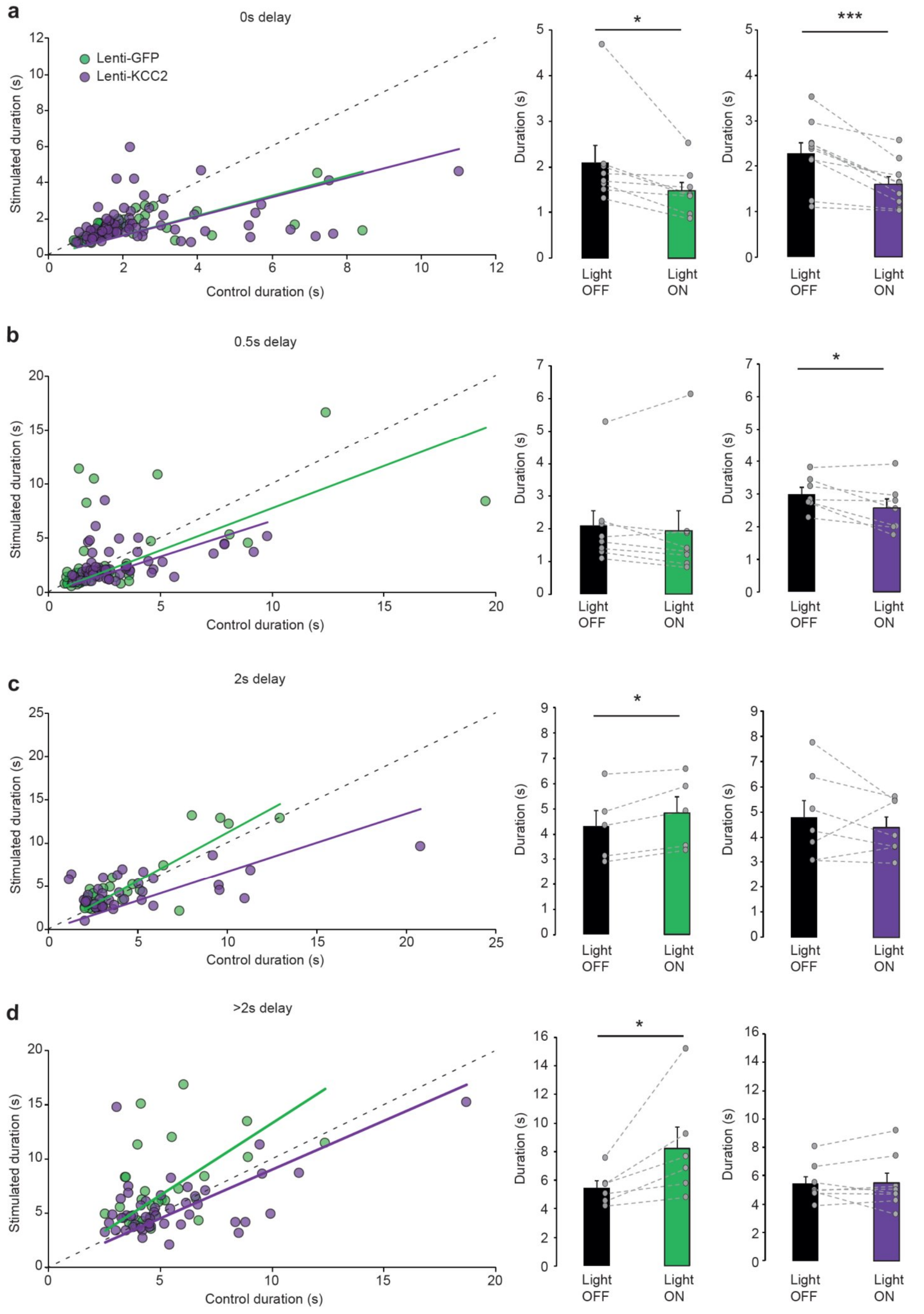

Supplementary Figure 7. Over-expression of the K+/Cl- co-transporter KCC2 in pyramidal neurons prevents the pro-seizure action of photo-depolarization of PV+ interneurons.

(a) Scatter plot (left): Effect of immediate (0 s delay) photo-depolarization of ChR2-expressing PV+ interneurons in mice either expressing GFP alone (*green*) or overexpressing KCC2 (*lilac*) in cortical pyramidal neurons. Each data point represents a pair of consecutive seizures, initially a control seizure and then one triggering laser activation (stimulated). The linear regression line (solid *green or lilac*) below the line of identity (*dashed black*) indicates a reduction in seizure duration by laser activation. The bar charts (right) show the absolute durations of control seizures and seizures triggering laser activation in GFP (n = 8 mice) and KCC2 (n = 10 mice) groups.

(b), (c), (d) Same as (a) but for 0.5s (GFP n = 8, KCC2 n = 7 mice), 2s (GFP n = 5, KCC2 n = 7 mice) and >2s (GFP n = 6, KCC2 n = 8 mice) delays respectively.

Paired t-test, * $p < 0.05$, ** $p < 0.01$; error bars represent s.e.m. Source data are provided as a Source Data file.
