# Supplementary material for: KCC2 overexpression prevents the paradoxical seizure-promoting action of somatic inhibition
Source: Nat Commun. 2019 Mar 15;10:1225. doi: 10.1038/s41467-019-08933-4 (PMC6420604; doi:10.1038/s41467-019-08933-4)
Supplement: Supplementary file 3 — Description of Additional Supplementary Files [file 41467_2019_8933_MOESM3_ESM.pdf]

Description of Additional Supplementary Files

File Name: Supplementary Movie 1
Description: **Simultaneous ECoG and video recorded during pilocarpine-induced seizures.** Simultaneous ECoG and video recordings were made during seizures induced by a pilocarpine injection into the mouse primary visual cortex. The animal is having motor manifestations during short ictal discharges; prolonged seizure activity is accompanied by rearing.
