# Supplementary material for: KCC2 overexpression prevents the paradoxical seizure-promoting action of somatic inhibition
Source: Nat Commun. 2019 Mar 15;10:1225. doi: 10.1038/s41467-019-08933-4 (PMC6420604; doi:10.1038/s41467-019-08933-4)
Supplement: Supplementary file 5 — Reporting Summary [file 41467_2019_8933_MOESM5_ESM.pdf]

nature research

Corresponding author(s): Ivan Pavlov, Vincent Magloire

Reporting Summary

Nature Research wishes to improve the reproducibility of the work that we publish. This form provides structure for consistency and transparency in reporting. For further information on Nature Research policies, see Authors & Referees and the Editorial Policy Checklist.

Statistical parameters

When statistical analyses are reported, confirm that the following items are present in the relevant location (e.g. figure legend, table legend, main text, or Methods section).

n/a	Confirmed	
☐	☒	The exact sample size (*n*) for each experimental group/condition, given as a discrete number and unit of measurement
☐	☒	An indication of whether measurements were taken from distinct samples or whether the same sample was measured repeatedly
☐	☒	The statistical test(s) used AND whether they are one- or two-sided<br>*Only common tests should be described solely by name; describe more complex techniques in the Methods section.*
☒	☐	A description of all covariates tested
☐	☒	A description of any assumptions or corrections, such as tests of normality and adjustment for multiple comparisons
☐	☒	A full description of the statistics including central tendency (e.g. means) or other basic estimates (e.g. regression coefficient) AND variation (e.g. standard deviation) or associated estimates of uncertainty (e.g. confidence intervals)
☐	☒	For null hypothesis testing, the test statistic (e.g. *F*, *t*, *r*) with confidence intervals, effect sizes, degrees of freedom and *P* value noted<br>*Give P values as exact values whenever suitable.*
☒	☐	For Bayesian analysis, information on the choice of priors and Markov chain Monte Carlo settings
☒	☐	For hierarchical and complex designs, identification of the appropriate level for tests and full reporting of outcomes
☒	☐	Estimates of effect sizes (e.g. Cohen's *d*, Pearson's *r*), indicating how they were calculated
☐	☒	Clearly defined error bars<br>*State explicitly what error bars represent (e.g. SD, SE, CI)*

Our web collection on statistics for biologists may be useful.

Software and code

Policy information about availability of computer code

Data collection	In vitro data acquisition software: WinEDR version 3.2.7 (University of Strathclyde, Glasgow), pClamp 10 (Molecular devices), LabView (National Instruments).<br>In vivo data acquisition: WinEDR version 3.2.7 (University of Strathclyde, Glasgow) in combination with LWDAQ software (Open Source Instrument, Inc). Laser was triggered using a LabView (National Instruments) routine.
Data analysis	In vitro and in vivo data were analysed using: WinEDR version 3.2.7 (University of Strathclyde, Glasgow), Clampfit 10 (Molecular devices) and a custom code in Python.

For manuscripts utilizing custom algorithms or software that are central to the research but not yet described in published literature, software must be made available to editors/reviewers upon request. We strongly encourage code deposition in a community repository (e.g. GitHub). See the Nature Research guidelines for submitting code & software for further information.

April 2018

Data

Policy information about availability of data

All manuscripts must include a data availability statement. This statement should provide the following information, where applicable:
- Accession codes, unique identifiers, or web links for publicly available datasets
- A list of figures that have associated raw data
- A description of any restrictions on data availability

The datasets generated and/or analysed during the current study are available from the corresponding authors on reasonable request. All figures have associated raw data.

Field-specific reporting

Please select the best fit for your research. If you are not sure, read the appropriate sections before making your selection.

☒ Life sciences ☐ Behavioural & social sciences ☐ Ecological, evolutionary & environmental sciences

For a reference copy of the document with all sections, see nature.com/authors/policies/ReportingSummary-flat.pdf

Life sciences study design

All studies must disclose on these points even when the disclosure is negative.

Sample size	Pilot experiments were performed in 2-3 animals to provide a rationale for the sample size
Data exclusions	Network state classifier was used to only select ictal-like activity in our analysis and to exclude other types of epileptiform discharges and mixed states (see Methods).
Replication	Control experiment on Figure 5 (CaMKII-GFP) replicates the main finding shown Figure 3.
Randomization	The delays of photostimulation was randomised in every experiment.
Blinding	All analyses of seizure durations were performed blind to the optogenetic stimulation.

Reporting for specific materials, systems and methods

Materials & experimental systems

n/a	Involved in the study
☒	☐ Unique biological materials
☐	☒ Antibodies
☒	☐ Eukaryotic cell lines
☒	☐ Palaeontology
☐	☒ Animals and other organisms
☒	☐ Human research participants

Methods

n/a	Involved in the study
☒	☐ ChIP-seq
☒	☐ Flow cytometry
☒	☐ MRI-based neuroimaging

Antibodies

Antibodies used	1.Polyclonal guinea pig anti-GFP antibody (Synaptic Systems Cat# 132 005 RRID: AB_11042617, 1:1000)<br>2.Polyclonal rabbit anti-RFP antibody (Rockland Cat# 600-401-379 RRID: AB_2209751, 1:500)<br>3.Donkey anti-guinea antibody conjugated with an alexa fluor 488 (A11073, lot 1737010, 1:1000)<br>4.Donkey anti-rabbit alexa fluor conjugated with an alexa fluor 568 (A10042, lot 1757124, 1:1000)
Validation	1.https://www.sysy.com/products/gfp/facts-132005.php<br>2.https://rockland-inc.com/store/Antibodies-to-GFP-and-Antibodies-to-RFP-600-401-379-O4L_24299.aspx<br>3 & 4 were secondary antibodies, control slices were incubated in the absence of the primary antibody to determine the background fluorescence.

Animals and other organisms

Policy information about studies involving animals; ARRIVE guidelines recommended for reporting animal research

Laboratory animals	PV::Cre (B6;129P2-Pvalbtm1(cre)Arbr/J, RRID: IMSR_JAX:008069) both sexes, aged between P60-120.<br>SOM::Cre (B6N.Cg-Ssttm2.1(cre)Zjh/J, RRID: IMSR_JAX:013044) both sexes, aged between P60-120.<br>C57BL/6 from Envigo aged P60-120 both sexes were used for experiments in Figure 4.
Wild animals	*Provide details on animals observed in or captured in the field; report species, sex and age where possible. Describe how animals were caught and transported and what happened to captive animals after the study (if killed, explain why and describe method; if released, say where and when) OR state that the study did not involve wild animals.*
Field-collected samples	*For laboratory work with field-collected samples, describe all relevant parameters such as housing, maintenance, temperature, photoperiod and end-of-experiment protocol OR state that the study did not involve samples collected from the field.*
